# 

**Published:** 1829-07-01

**Authors:** 


### CONTENTS

OF THE

### MEDICO-CHIRURGICAL REVIEW.

No. XXI. JULY 1, 1829.

### REVIEWS.

I.

Pathological and Practical Researches on Diseases of the Intestinal Canal. By JOHN ABERCROMBIE, M.D. .. 1

II.

An Essay on the Operation of Poisonous Agents on the Living Body. By Dr. ADDISON and Mr. MORGAN .. 32

III.

On Aneurism and its Cure by a New Operation. By JAMES WARDROP, Esq. .. 46

IV.

DUTROCHET's Discoveries in Vegetable and Animal Physiology .. 49

V.

The Medical Calendar; or Student's Guide to the Medical Schools, &c. .. 70

VI.

Dr. MILLS on the Morbid Appearances in Disorders of the Trachea, Lungs, and Heart, &c. .. 75

VII.

An Essay on the Mechanism of Parturition .. 88

VIII.

The Study of Medicine. By J. M. GOOD, M,D. edited by Mr. S. COOPER .. 91

IX.

The Morbid Anatomy of the Bowels, Liver, and Stomach, &c. By JOHN ARMSTRONG, M.D.—Fasciculus III. .. 97

X.

Engravings of the Nerves, copied from the Works of Scarpa, &c. By Dr. KNOX.. 108

XI.

A Treatise on Obstructed and Inflamed Hernia. By HENRY STEPHENS, M.R.C.S. 109

XII.

A System of Human Anatomy, from the French of M. Cloquet. By Dr. KNOX .. 117

XIII.

Dr. CLARK on the Influence of Climate in the Prevention and Cure of Diseases .. 121

XIV.

Mr. MADDEN's Travels in Turkey, Egypt, Nubia, and Palestine .. 141

### PERISCOPE.

1. Curious Cases of Dislocation of the Patella outwards .. 145
2. Observations on the Treatment of Small-Pox. By A. Stewart, Esq. .. 148
3. Hypochondriasis as dependent on Chronic Gastritis and Enteritis .. 150
4. The Leaping Ague of Angus .. 151
5. Provincial Medical Gazette—Measure for Measure—Medical Politics at a County Fire-side .. 152
6. Phlebitis after Venesection .. 155

7. Aneurism of the Brachial Artery from Venesection .. .. .. .. .. .. .. 157
8. Imperfection of the Heart—Discoloration of the Skin—Post-mortem Appearances .. .. .. .. .. .. .. .. .. .. .. .. .. .. .. .. .. .. 159
9. On the Moral and Physical Character (Cerebral Development) of Burk.. .. 193
10. Helleborus Albus used for producing Artificial Cardiac Disease .. .. .. 196
11. Corporation Monopolies in Ireland.. .. .. .. .. .. .. .. .. .. .. .. 196
12. Charge of Murder—Knife found in the Abdomen of a Female .. .. .. .. 199
13. Bloody Tumour of Bone .. .. .. .. .. .. .. .. .. .. .. .. .. .. .. 200
14. Dr. Gordon on Clinical Medicine .. .. .. .. .. .. .. .. .. .. .. .. 201
15. On Union by the first Intention .. .. .. .. .. .. .. .. .. .. .. 202
16. On Medical Reporting .. .. .. .. .. .. .. .. .. .. .. .. .. .. 204
17. Observations on the Polish Carbuncle .. .. .. .. .. .. .. .. .. .. 204
18. Extirpation of the Tonsil .. .. .. .. .. .. .. .. .. .. .. .. .. 208
19. On Catarrhus Vesicæ.. .. .. .. .. .. .. .. .. .. .. .. .. .. .. 209
20. Dr. Hood on the External Application of Nitrate of Silver in various Diseases 211
21. Case of Masked Double Tertian .. .. .. .. .. .. .. .. .. .. .. 215
22. Mr. Stafford on Urethral Strictures .. .. .. .. .. .. .. .. .. .. 216
23. Mr. Warburton's Bill .. .. .. .. .. .. .. .. .. .. .. .. .. .. 218
24. Remarkable Case of various unsuspected Lesions, ending in Atrophy. By Dr. Johnson .. .. .. .. .. .. .. .. .. .. .. .. .. .. .. .. .. 241
25. Case of Internal Strangulation that led to a Suspicion of Poison .. .. .. 247
26. On Acute Idiopathic Carditis .. .. .. .. .. .. .. .. .. .. .. .. 248
27. On False Aneurism of the Heart .. .. .. .. .. .. .. .. .. .. .. 249
28. Italian Practice, by Antimony, anticipated by Dr. Marryatt .. .. .. .. 253
29. Dr. Thomson's New Theory of Digestion .. .. .. .. .. .. .. .. .. 254
30. Ligature of the Spermatic Artery for Varicocele .. .. .. .. .. .. .. 256
31. Experiments on the Operation of Morphine in the Human Body .. .. .. 257
32. Rapid Development of an Ovarian Disease.. .. .. .. .. .. .. .. .. 258
33. Case of Amnesia, or Loss of Language .. .. .. .. .. .. .. .. .. .. 259

### CLINICAL REVIEW.

WINCHESTER COUNTY HOSPITAL .. .. .. .. .. .. 161

1. Paralysis of the Upper Extremity .. .. .. .. .. .. .. .. .. .. .. .. 163

2. Amputation and Consequences .. .. .. .. .. .. .. .. .. .. .. .. 163

3. Spontaneous Cure of Femoral Aneurism .. .. .. .. .. .. .. .. .. .. 164

4. Wound of the Heart .. .. .. .. .. .. .. .. .. .. .. .. .. .. .. 165

5. Food in the Pharynx for a Year .. .. .. .. .. .. .. .. .. .. .. 166

6. Dissection of a Case of Ascites.. .. .. .. .. .. .. .. .. .. .. .. 266

7. Extensive Disease of the Kidneys and Bladder .. .. .. .. .. .. .. .. 267

8. Sudden Protrusion of the Intestines into the Scrotum.. .. .. .. .. .. .. 268

HÔTEL DIEU DE CAEN.

1. Acupuncturation .. .. .. .. .. .. .. .. .. .. .. .. .. .. .. .. .. 166

MADRAS GENERAL HOSPITAL.

1. Acute Hepatitis .. .. .. .. .. .. .. .. .. .. .. .. .. .. .. .. .. 167

MAISON ROYALE DE LA LEGION D'HONNEUR.

1. Epidemic Croup .. .. .. .. .. .. .. .. .. .. .. .. .. .. .. .. 169

BALTIMORE INFIRMARY.

1. Arthrosia Atonica .. .. .. .. .. .. .. .. .. .. .. .. .. .. .. .. 170

La Charite.

1. On the Excision of Nævi Materni .. .. .. .. .. .. .. .. .. .. .. 172

2. Injection of the Tunica Vaginalis, &c. .. .. .. .. .. .. .. .. .. .. 174

3. Abscess between the Vagina and Rectum .. .. .. .. .. .. .. .. .. 175

4. Angina Œdematosa .. .. .. .. .. .. .. .. .. .. .. .. .. .. 175

5. Observations on Neuralgic Affections of the Genito-Urinary Organs.. .. .. 276

Glasgow Royal Infirmary.

1. Strangulated Hernia .. .. .. .. .. .. .. .. .. .. .. .. .. .. 176
2. Hydrocele of the Spermatic Cord complicated with Peritonitis .. .. .. .. 178
3. Nævus cured by Vaccination .. .. .. .. .. .. .. .. .. .. .. .. 269
4. Lumbar Abscess pointing behind the Trochanter .. .. .. .. .. .. .. 270
5. Sloughing of the Scrotum, with Cases .. .. .. .. .. .. .. .. .. .. 270
6. Partial Paralysis cured by Strychnine .. .. .. .. .. .. .. .. .. .. 272
7. Affection of the Leg resembling Elephantiasis .. .. .. .. .. .. .. 272

Fever Hospital, Cork Street, Dublin.

1. Medical Report of the House of Recovery and Fever Hospital, for the Year 1827. By John O'Reardon, M.D. .. .. .. .. .. .. .. .. .. .. .. 181

Royal Westminster Ophthalmic Hospital.

1. On Muscæ Volitantes. By Mr. Guthrie.. .. .. .. .. .. .. .. .. .. 184

St. George's Hospital.

1. Cases in which the Blood was found fluid after Death.. .. .. .. .. .. 185
2. Curious Morbid Condition of the Arachnoid Membrane of the Brain .. .. 187
3. Cases of Open Foramen Ovale... .. .. .. .. .. .. .. .. .. .. .. 230
4. Treatment of Nævi .. .. .. .. .. .. .. .. .. .. .. .. .. .. .. 232
5. On Injuries of the Head .. .. .. .. .. .. .. .. .. .. .. .. .. 232

Royal Infirmary of Edinburgh.

1. Dr. Ballingall on Fractures .. .. .. .. .. .. .. .. .. .. .. .. 226
2. On Primary Amputation, with Cases. Dr. Ballingall .. .. .. .. .. .. 279
3. On Secondary Amputation .. .. .. .. .. .. .. .. .. .. .. .. .. 281

Sunderland Dispensary.

1. Curious Case of Chorea .. .. .. .. .. .. .. .. .. .. .. .. .. 236

2. Case of Purpura Hæmorrhagica .. .. .. .. .. .. .. .. .. .. .. 237

Bath Hospital.

1. Injuries of the Head .. .. .. .. .. .. .. .. .. .. .. .. .. .. 238

Hôtel Dieu.

1. Treatment of imperforate Rectum .. .. .. .. .. .. .. .. .. .. .. 261
2. Treatment of Ranula, by Seton .. .. .. .. .. .. .. .. .. .. .. 263
3. Supposed Hernia of the Lungs from tight Stays .. .. .. .. .. .. .. 263
4. Hernia; Absence of Testicles from the Scrotum.. .. .. .. .. .. .. 263
5. Popliteal Aneurism—Gangrene—Death .. .. .. .. .. .. .. .. .. 264

Hôpital du Gros Caillou.

1. New Mode of treating Perineal Fistula .. .. .. .. .. .. .. .. .. 265

Worcester Infirmary.

1. Curious Case of Ischuria. By Dr. Hastings .. .. .. .. .. .. .. .. 274

Philadelphia Infirmary.

1. Malignant Intermittent cured without Bark.. .. .. .. .. .. .. .. 278

WESTMINSTER HOSPITAL.

1. Injuries of the Head .. .. .. .. .. .. .. .. .. .. .. .. .. .. .. .. .. .. 283

**MEDICAL JURISPRUDENCE.**

1. Plan of Amelioration of the Medical Profession, &c. By Alexipharmacus .. 189
2. Trial of Martin the Incendiary .. .. .. .. .. .. .. .. .. .. .. .. .. .. 221
3. Mr. Guthrie's Lectures at the Royal College of Surgeons .. .. .. .. .. .. 225

MISCELLANIES.

1. Defeat of the Anatomy-bill, with reflexions .. .. .. .. .. .. .. .. .. .. 285
2. Curious illustrations of St. John Long's Cures of Consumption, with obituary attestations .. .. .. .. .. .. .. .. .. .. .. .. .. .. .. .. .. .. 287
3. Bibliographical Record .. .. .. .. .. .. .. .. .. .. .. .. .. .. .. .. .. 288

